# Odor Memory Stability after Reinnervation of the Olfactory Bulb

**DOI:** 10.1371/journal.pone.0046338

**Published:** 2012-10-10

**Authors:** Eduardo Blanco-Hernández, Pablo Valle-Leija, Viviana Zomosa-Signoret, René Drucker-Colín, Román Vidaltamayo

**Affiliations:** 1 Departamento de Neuropatología Molecular, Instituto de Fisiología Celular, Universidad Nacional Autónoma de México, Distrito Federal, México; 2 Departamento de Bioquímica, Facultad de Medicina, Universidad Autónoma de Nuevo León, Nuevo León, México; 3 Departamento de Ciencias Básicas, Centro de Diagnóstico Molecular y Medicina Personalizada, Universidad de Monterrey, Nuevo León, México; Université Lyon, France

## Abstract

The olfactory system, particularly the olfactory epithelium, presents a unique opportunity to study the regenerative capabilities of the brain, because of its ability to recover after damage. In this study, we ablated olfactory sensory neurons with methimazole and followed the anatomical and functional recovery of circuits expressing genetic markers for I7 and M72 receptors (M72-IRES-tau-LacZ and I7-IRES-tau-GFP). Our results show that 45 days after methimazole-induced lesion, axonal projections to the bulb of M72 and I7 populations are largely reestablished. Furthermore, regenerated glomeruli are re-formed within the same areas as those of control, unexposed mice. This anatomical regeneration correlates with functional recovery of a previously learned odorant-discrimination task, dependent on the cognate ligands for M72 and I7. Following regeneration, mice also recover innate responsiveness to TMT and urine. Our findings show that regeneration of neuronal circuits in the olfactory system can be achieved with remarkable precision and underscore the importance of glomerular organization to evoke memory traces stored in the brain.

## Introduction

The olfactory system offers a unique opportunity to study the mechanisms of neuronal regeneration. Lying on the olfactory epithelia (OE), olfactory sensory neurons (OSNs) are responsible for the initial process of odorant detection. This neuronal population is replaced continuously during adult life [1], [2], [3]. Several studies have shown the remarkable capability of regeneration of OSNs in the OE and reinnervation of their postsynaptic targets in the olfactory bulb (OB) after diverse insults [4]–[7]. Moreover, the organization of neuronal circuits within the OE and OB allows for monitoring of anatomical and functional recovery after damage [1]. Each OSN expresses only one of ∼1000 possible olfactory receptors (ORs) [8]–[11]. All the OSNs expressing a specific OR innervate the same area of the OB, where their axons coalesce and form functional circuits called glomeruli.

For each population of OSNs, there is at least one glomerulus lying on the lateral side and one on the medial side of the OB [12]–[16]. This topographic organization of glomeruli is stereotyped among individuals [17]–[21]. Glomeruli constitute an anatomical feature that organizes the incoming sensory inputs to the OB: An odorant molecule activates a variety of ORs and every OR recognizes several chemically-related odorant molecules [22]–[25], but a specific set of glomeruli is activated by a particular odorant mixture [14], [26]–[31]. Hypothetically, these maps of glomerular activation are closely related to the subsequent neural processing that defines the identity and possibly the qualities of odor molecules [32], [33], [34]. Supporting this hypothesis, studies inducing the degeneration of the OE have shown that the precise glomerular organization is severely disrupted after re-innervation of the OB [1], [5], [35]–[37]. These alterations in the glomerular circuit correlate with loss of learned olfactory tasks [38], without affecting the basic function of detection and discrimination of odorants [39], [40]. However, it is not clear whether the loss of olfactory performance is caused by distorted glomerular maps, or due to memory loss produced by changes in circuitry after denervation of sensory fibers.

In this context, new models of OSN regeneration that allow a better recovery of the glomerular organization are necessary to clarify the role that glomerular activated maps have during perception of odorants and recall of memory tasks associated to those same odorants. Here, we used the anti-thyroid drug methimazole to induce degeneration of the OE in knock-in mice expressing genetic markers for the M72 and I7 receptors (M72-IRES-tau-LacZ and I7-IRES-tau-GFP). Contrary to other models of degeneration [6], methimazole preserves the integrity of the lamina propria (LP) and cribriform plate, which are essential for sensory axon fasciculation and extension during re-innervation of the bulb. We analyzed the regenerative capability of OSN populations as well as the precision of glomerular re-innervation, and examined the functional implications of glomerular circuitry regeneration for learned and innate olfactory behavior.

## Results

### M72 circuits are restored after methimazole treatment

**Figure 1:**
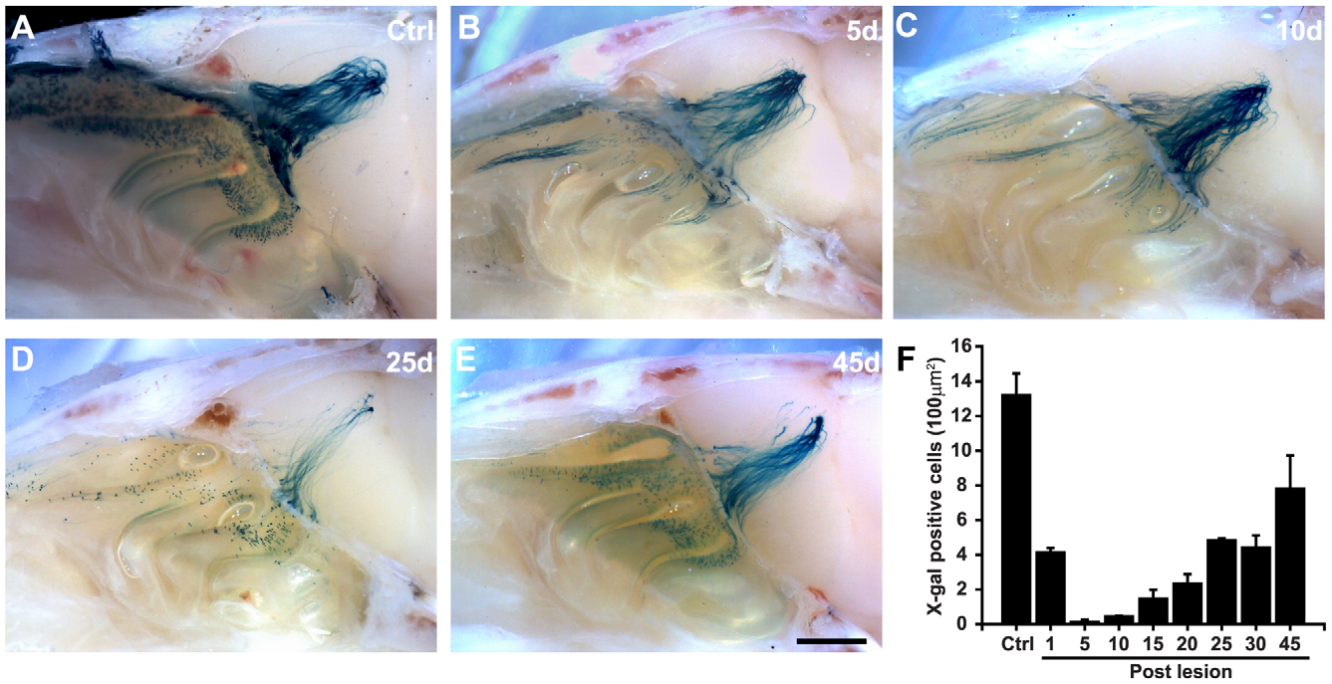
M72 olfactory circuit is restored following ablation by methimazole administration. **A**, Medial view of OE and OB is shown. M72 neurons lie at the dorsal portion of the turbinates, their axons innervate the dorsal aspect of OB were they coalesce into glomeruli. **B**, No cell bodies can be observed 5 days post lesion. **C**, Ten days after lesion few M72-positive neurons are observed. X-gal stained axons and glomeruli remained visible on the surface of the bulb. **D**, 25 days post lesion M72-positive neurons could be observed in the turbinates and their axons have re-innervated the OB. **E**, After 45 days, M72-positive cell bodies and glomeruli have regenerated almost completely. The pattern of innervation is similar to control animals even though some wandering axons remain visible. **F**, Cell counts at the turbinates show the time-course of regeneration of X-gal stained cells. Scale bar 500 µm. Mean ± SEM are represented.

The temporal course of axonal regeneration of neurons expressing the M72 receptor was followed during 45 days after methimazole administration. Figure 1 shows the medial aspect of the nasal cavity and olfactory bulb of M72-IRES-tauLacZ mice. These M72-expressing OSNs are located in the dorsal portion of the nasal turbinates. Their axons project to the dorsal aspect of the olfactory bulb, where they coalesce into glomeruli [43], [44]. Virtually all M72-positive OSNs were ablated five days after methimazole administration (Fig. 1F). Ten days after methimazole exposure almost no M72-positive cell bodies could be detected on the surface of the turbinates (Fig. 1B), while blue-stained axon fibers remained visible on the OB. The number of M72-expressing neurons gradually recovered after methimazole administration (Fig. 1F, see below). The distribution of M72-positive neurons on the dorsal part of the turbinates was reestablished, similarly to what has been reported with other methods of OE ablation [45]. Strikingly, at 45 days post-lesion, the pattern of projection of the M72 circuit was qualitatively similar to the control condition, even though some axons were located off-target (Fig. 1E).

### The entire population of M72 neurons is recovered after methimazole treatment

**Figure 2:**
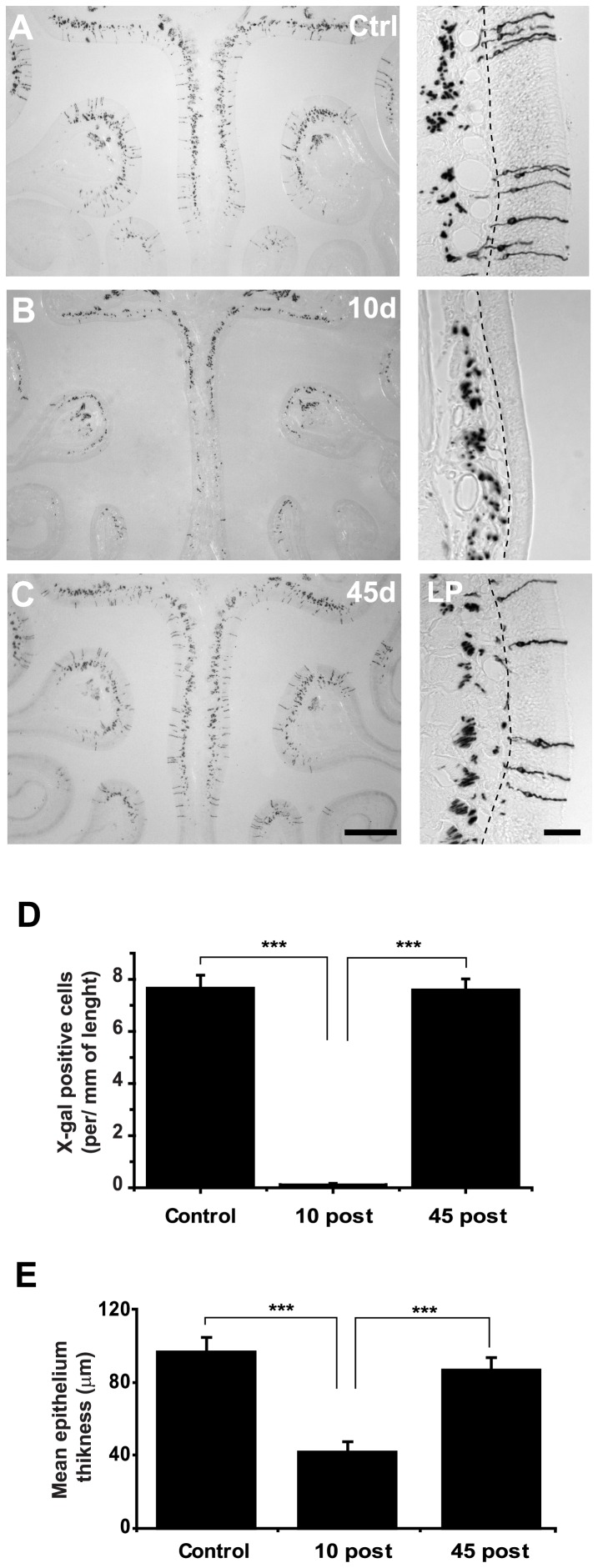
M72-expressing OSN population is regenerated 45 days after methimazole treatment. **A**, Coronal view of control olfactory epithelium is shown. At higher magnification X-gal stained neurons can be observed within the OE and axons can also be seen traversing the LP. **B**, Almost no X-gal stained neurons can be observed 10 days post lesion. Some axons bundles remain visible along the LP. **C**, 45 days after methimazole induced lesion, the regenerated olfactory epithelium cannot be distinguished from that of control animals. **D**, Cell counts in the OE show that the population of M72 neurons is regenerated after methimazole treatment. **E**, Thickness of olfactory epithelia is significantly decreased 10 days post lesion. Mean ± SEM. Scale bars 400 µm and 80 µm in OE magnifications. *** denotes *P*<0.05.

To accurately determine the extent of OSNs loss and recovery after methimazole treatment, we evaluated OE histological sections from M72-IRES-tau-LacZ mice to quantify X-gal stained neurons. Figure 2 shows representative images of coronal sections of the olfactory turbinates at 10 and 45 days post lesion. Ten days after methimazole exposure, nearly all M72-positive neurons were eliminated (7.6±0.5 vs. 0.1±0.05 cells/mm length, control vs.10 days post lesion; mean ± SEM; *P*<0.0001, Fig. 2B). Remarkably, X-gal stained axons remained visible within the *lamina propria* (Fig. 2B right panel), confirming what we observed in the whole mount preparations. The entire M72-positive population is renewed in the OE 45 days after methimazole treatment (7.6±0.5 vs.7.5±0.4, cells/mm length, control vs. 45 days post lesion; *P*>0.05, Fig. 2D). We also measured the thickness of olfactory epithelia and observed a significant decrease 10 days post-lesion (96.67±4.8 vs. 42.04±1.2 µm, control and 10 days post-lesion; *P*<0.0001, Fig. 2E). No statistical difference in thickness was found 45 days post-lesion (96.67±4.8 vs. 87.06±2.8 µm, control vs. 45 days post-lesion; *P*>0.05). No evidence of metaplasia or damage to the underlying structures of the epithelia was observed 45 days post-ablation.

### Location of M72 glomeruli is restored after regeneration

**Figure 3:**
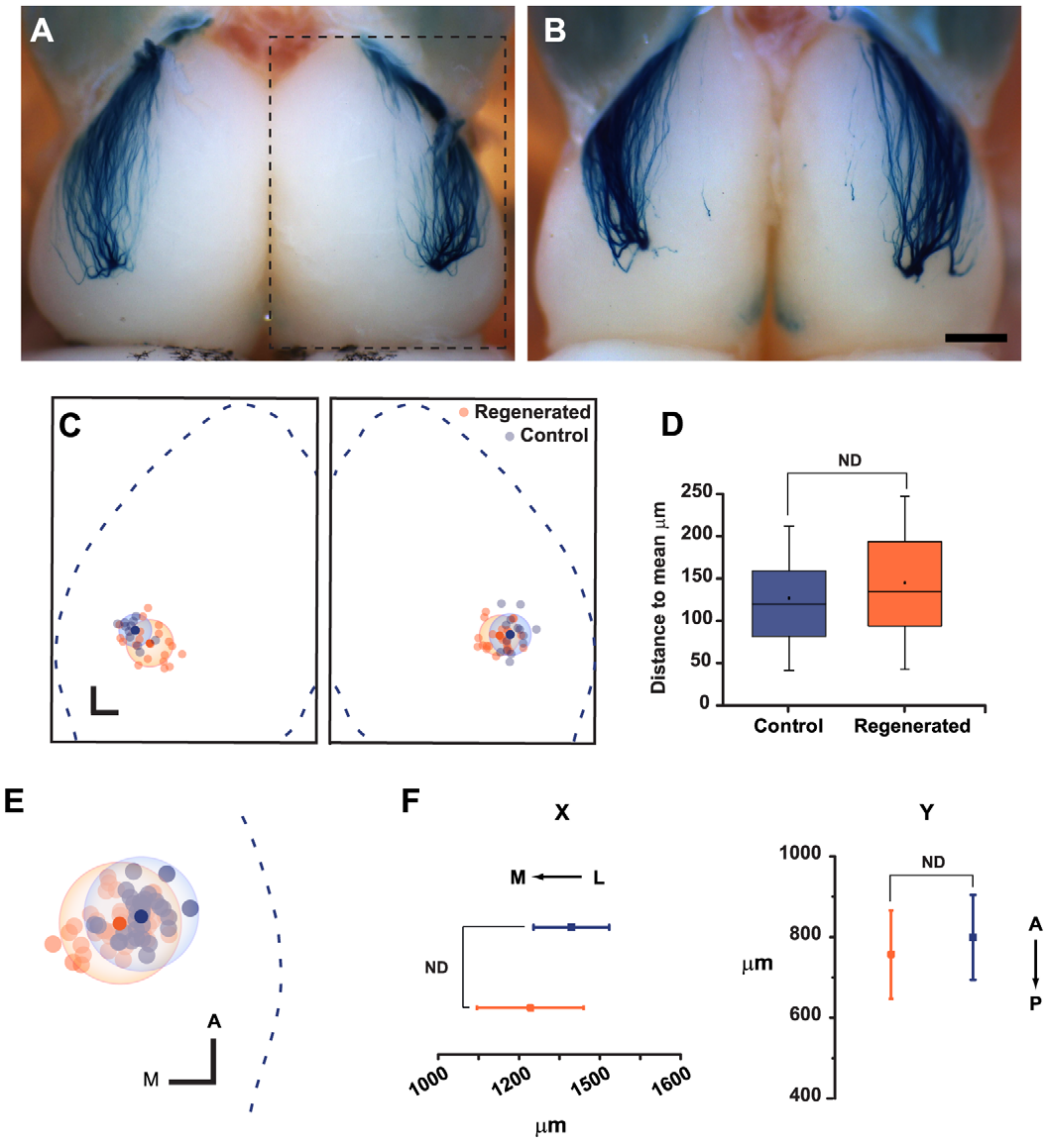
Position of M72 lateral glomerulus is recovered after regeneration. **A**, Image of the dorsal aspect of a M72-IRES-tauLacZ control mouse showing the lateral glomeruli on the surface of OB. **B**, Representative image of M72 lateral glomeruli 45 days after methimazole administration. Notice the striking similar pattern of innervation compared to control animals. **C**, Summary of glomerular distribution after regeneration. The normalized dorsal aspect of olfactory bulbs is represented (dashed line in A shows the OB contour taken in every image to perform the normalization), the centroid of all glomeruli in control animals (blue dots) and 45 days post lesion (orange dots). The mean position (filled dots) and 2 standard distances (transparent spheres around the means) are shown. **D**, Box plot of the values of Euclidean distances showing no significant differences between control and regenerated mice. **E**, All glomeruli projected on the right plane at a higher magnification. **F**, The analysis of the mean position carried on the normalized X- and Y-axes shows no significant differences in glomerular location following regeneration. Mean ± SD are represented. Scale bars 500 µm (C) and 200 µm (D, F). Latero-medial (L–M), Antero-posterior (A–P), No difference (ND).

In order to refine our observations on the projection patterns of these regenerated circuits, we performed spatial analysis of the position of glomerular structures on the dorsal portion of the OB from control and regenerated mice. Figure 3 shows representative images of the dorsal aspect of the olfactory bulb of control (3A) and regenerated mice (3B). Forty-five days after methimazole treatment, the innervation pattern of regenerated M72 axons to the olfactory bulb was very similar to the one observed in control animals. To better describe the position of glomeruli after regeneration, images were normalized to the average size of the OB (see Materials and Methods). Figure 3C shows normalized bulbs and the position of glomeruli observed in control (blue dots) and methimazole-treated mice (orange dots). First, we assessed differences in projection precision by analyzing the dispersion of glomeruli around the mean position by use of Euclidean distance measures (see Materials and Methods). As shown in figure 3D, there were no significant differences in the variance of the Euclidean distance to the mean glomerular position between both animal groups (*P*>0.05). To evaluate differences in projection accuracy, the mean position of regenerated glomeruli was examined. Figure 3E shows the summary of the relative position of all glomeruli projected on a single virtual plane representing the dorsal portion of the right hemibulb (images from the left hemibulbs were mirror-inverted digitally and projected on the same plane for analysis purposes). There were no significant differences in glomerular positions between control and regenerated animals (n = 32 and n = 42, respectively) (Fig. 3F, *P*>0.05).

**Figure 4:**
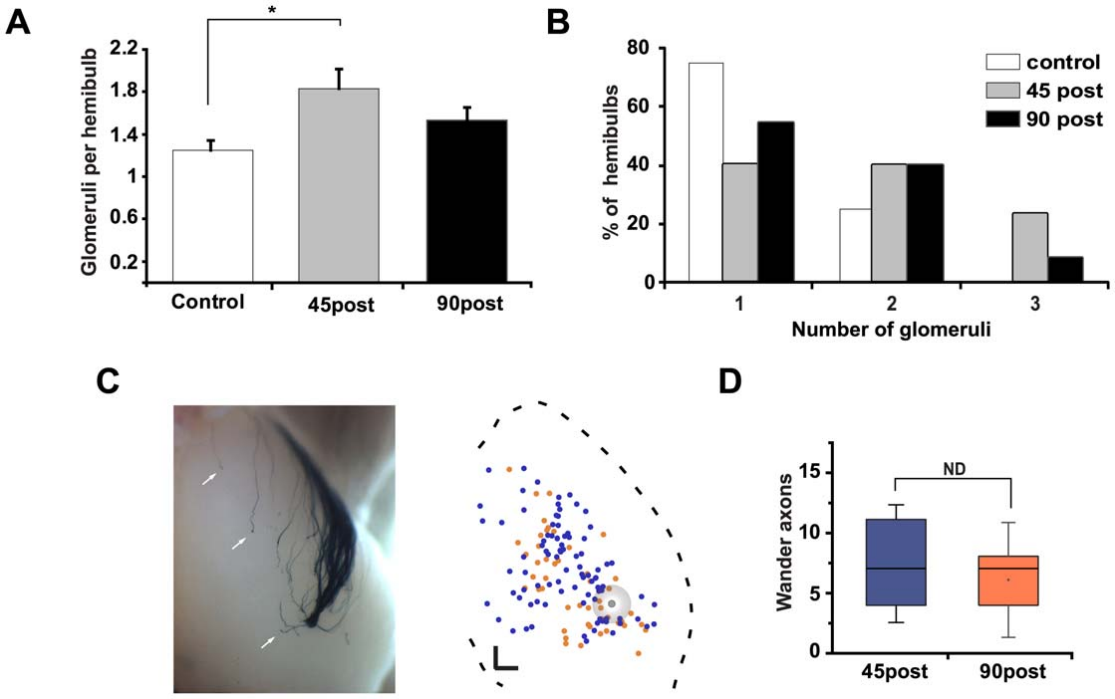
No refinement of glomerular circuits after regeneration. **A**, The mean number of lateral glomeruli increased 45 days after methimazole administration (* denotes *P*<0.05). No evident refinement of glomeruli is observed 90 days after methimazole exposure (*P*>0.05). **B**, Percentage of hemibulbs showing the indicated number of glomeruli. **C**, Image of dorsal bulb from regenerated mice (left) and normalized plane with the distribution of miss routed axons at 45 (blue dots) and 90 (orange dots) days post lesion (right). **D**, Box plot of the number of miss routed axons. Mean ± SEM are represented. Scale bar 200 µm, (ND, no difference).

On the other hand, we observed a small but significant, increase in the number of M72 glomeruli present on the dorsal surface of regenerated bulbs 45 days after methimazole exposure (1.24±0.4 vs.1.8±0.7, glomeruli/hemibulb, control vs. regenerated animals; *P*<0.05, Fig. 4A). This increased number of glomeruli persisted 90 days after methimazole injection, contrasting with the refinement process observed during neonatal development, where supernumerary glomeruli tend to disappear over time [46]. The frequency distribution of lateral glomeruli per hemibulb illustrates an increase in glomeruli numbers (Fig. 4B). To further explore the refinement of mistargeted projections to the bulb after regeneration, we analyzed the position of those axons on the dorsal OB. Figure 4C shows a representative image of regenerated, mistargeted M72 axons, most of them single fibers not coalescing with other major M72-positive glomeruli (arrows). In overlapped images, obtained 45 and 90 days post- methimazole ablation, no apparent differences in the location patterns and axon numbers were observed (figure 4C right and 4D).

### I7 circuits are also restored after methimazole administration

**Figure 5:**
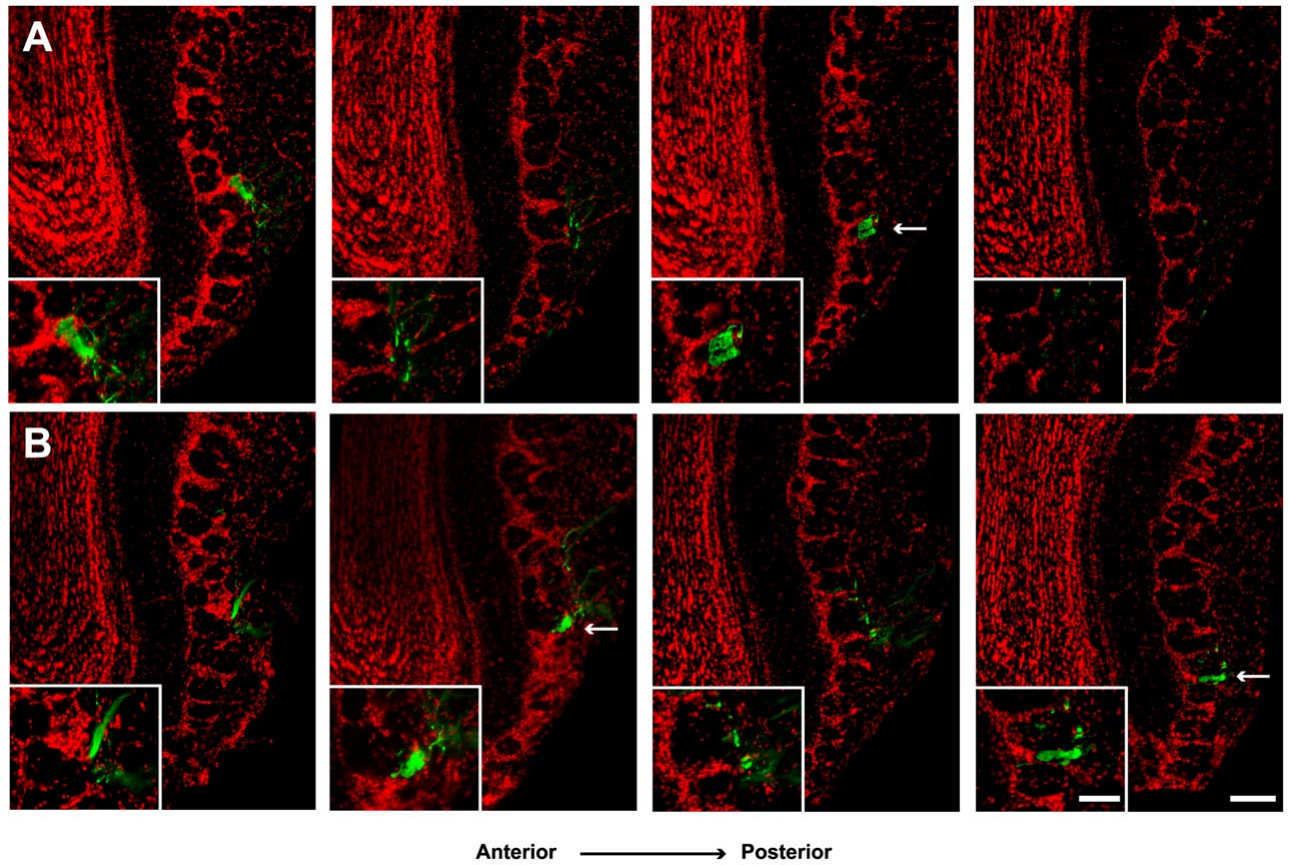
I7 glomerular circuit is restored 45 days after methimazole-induced lesion. Representative images of serial coronal slices of bulbs from I7-IRES-tauGFP mice are shown. **A**, The projection of GFP-positive axons into the antero-ventro-lateral aspect of control OB, it can be observed that they coalesce in one lateral glomerulus (arrow). **B**, 45 days post-lesion the axons project mainly into two adjacent locations (arrows) in the same area shown in A. Insets at the bottom show a close-up of GFP fibers. Space between slices is 40 µm. Scale bar 100 µm and 50 µm for insets.

To determine if our findings on olfactory circuit regeneration were restricted to the M72 population or, on the other hand, could be observed in other areas of the bulb, we studied the degree of regeneration of the I7 glomerular circuit located on the ventral region of the OB [24], [25]. We analyzed seven mice, 3 in the control and 4 in the experimental group, expressing the histological genetic marker tauGFP in I7-positive OSNs (I7-IRES-tauGFP). The regenerated projections to glomeruli were studied 45 days after methimazole injection. Figure 5 shows representative images of serial, coronal OB slices OB from age-matched control animals (Fig. 5A) and 45 days after methimazole-exposure (Fig. 5B). Images show serial sections of the anteroventral region of the OB (see methods) where the lateral glomerulus is located. Projections of GFP positive axons in control mice innervated this area and formed one single glomerulus (arrow in Fig. 5A and inset). After regeneration, the GFP-positive axons projected to the same area and entered in to the glomerular layer similarly to control animals. However, axons innervated two adjacent glomeruli (arrows in Fig. 5B, and inset). Also innervation to some glomeruli appeared irregular (Fig. 5B, arrow right), since the GFP fibers did not fill the entire glomeruli. The same projection pattern was observed consistently in all tested mice.

### A learned odor-guided task is recalled after regeneration

**Figure 6:**
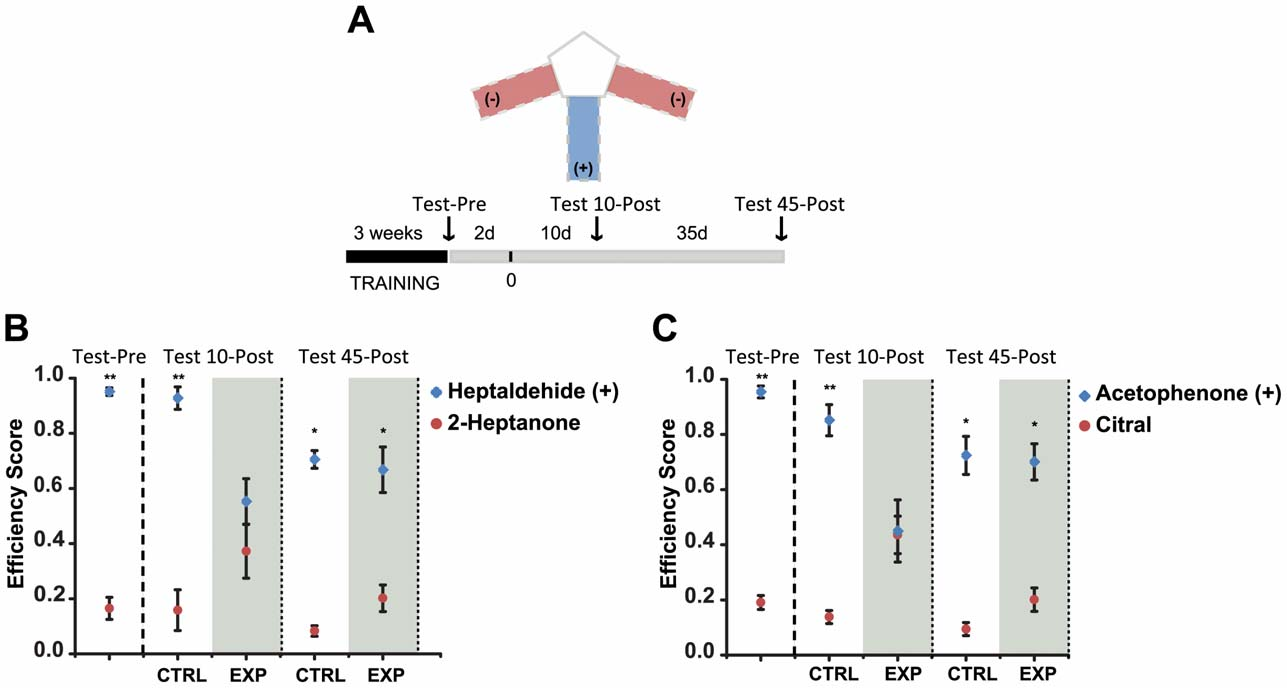
Memory of an olfactory discrimination task is recovered after the regeneration. **A**, Diagram of the three-arm maze and temporal course of the experiments is shown. **B** and **C**, Performance in the discrimination task pre- and post-methimazole administration in group 1 and group 2 respectively. Both groups discriminate the odorants after training (Test-Pre) (** denotes *P*<0.01). Ten days after methimazole treatment the experimental group (EXP) fails to perform the task (*P*>0.05), 45 days post lesion, mice recover the ability to perform the task (* denotes *P*<0.05). Mean ± SEM are represented.

We evaluated the extent of functional recovery and preservation of odor-dependent memories following reinnervation by a learned odor-discrimination task. We focused in odor molecules known to activate glomerular circuits in diverse zones along the OB including the cognate ligands for M72 and I7 receptors (acetophenone and heptaldehyde, respectively) [24], [47]. Twenty-four mice were randomly separated into two groups and each group was tested with different pairs of odorants, acetophenone vs citral (Group 1) or heptaldehyde vs 2-heptanone (Group 2). The task consisted in associating the presence of given odorant in the environment with receiving water as a reward (details in Materials and Methods). Figure 6A illustrates a diagram of the maze and the experimental design. Figures 6B and 6C show the performance of Group 1 and Group 2 in both (blue dots represent data from the rewarded arm of the maze (i.e. the zone with the water reward and the specific odorant; +arm, blue dots) and the not-rewarded arm (−arm, red dots). Control groups retained the ability to discriminate the odorants even when tested 45 days after finalizing the training period (Group 1: 0.7±0.04 vs. 0.12±0.03, efficiency score in +arm vs. −arm; *P*<0.05. Group 2: 0.7±0.03 vs. 0.8±0.01; *P*<0.05). Animals from the methimazole-treated (EXP) group were unable to discriminate the different odorants 10 days after injection (Group 1: 0.45±0.1 vs. 0.43±0.2, efficiency score in +arm vs. −arm; *P*>0.05. Group 2: 0.57±0.09 vs. 0.37±0.09; *P*>0.05). This observation correlates well with the absence of olfactory sensory neurons in the OE at 10 days after methimazole injection (Fig. 2). Remarkably, the EXP Group recovered the ability to discriminate the odorants 45 days after lesion (Group 1: 0.7±0.06 vs. 0.2±0.04, efficiency score in +arm vs. −arm; *P*<0.05. Group 2: 0.60±0.08 vs. 0.2±0.04; *P*<0.05).

### Innate odor-dependent behavior is partially recovered after regeneration

Previous studies have shown that the main OB mediates the innate response to some odorants, such as the aversive response to Trimethyl-thiazoline (TMT, a compound present in fox feces [41]) and the attractant response to urine [47], [48]. Thus, we decided to further explore the functional recovery after methimazole treatment by evaluating the innate behavior elicited by these odorants in a preference task. We hypothesized that reestablishment of glomerular circuit organization following methimazole-induced ablation could lead to recovery of innate responses to odorants.

**Figure 7:**
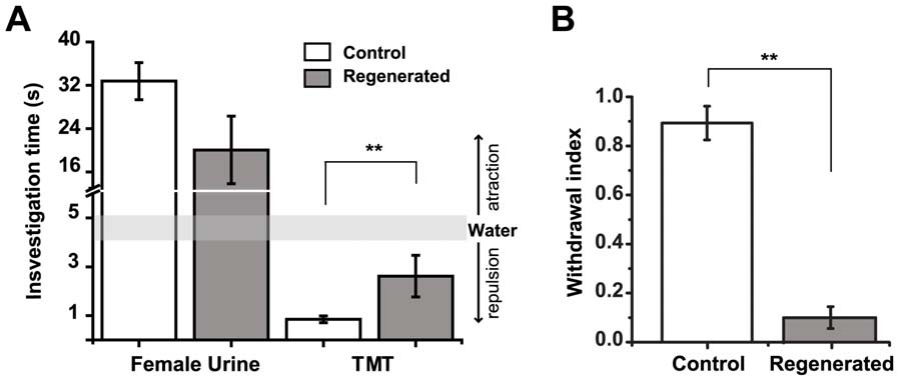
Odorant-clued innate behavior is partially recovered after regeneration. **A**, Histogram shows the investigation time for female urine and TMT. Behavioral responses to attractive female urine from regenerated males cannot be distinguished from control animals 45 days after OE ablation (*P*>0.05). However, responses to the repulsive odorant TMT are significantly different between control and regenerated animals. The latter showing significant increases in investigation time of TMT-scented filter paper and **B**, decreased withdrawal responses to this pheromone 45 days after ablation. (** denotes *P*<0.01). Mean ± SEM are represented.

Mice were exposed to TMT or urine –scented filter paper and the animal's investigation time was measured during 3-min periods. Figure 7A shows the investigation time for the different odorants 45 days after methimazole exposure and from age-matched control male mice. Female urine elicited strong attraction in control as well as in regenerated mice (33.4±2.8 vs.19.1±5.1 s, control vs. regenerated animals; *P*>0.05, n = 6 per group). Also, we observed that they were able to mate with no evident problems (data not shown). In contrast, although TMT elicited repulsive behavior in regenerated mice (Figure 7A), the investigation time was significantly increased in regenerated mice (0.8±0.1 vs. 2.4±0.7 s, control vs. regenerated animals; *P*<0.01, n = 6 per group). Moreover, withdrawal index (times mouse head abruptly changed direction/times the mouse approached the stimulus) from TMT was drastically decreased in regenerated mice (0.89±0.06 vs. 0.1±0.04 s, control vs. regenerated mice; *P*<0.01, n = 6 per group. Figure 7B).

## Discussion

This study shows for the first time that the process of regeneration after extensive damage of the OSNs is precise enough to restore the major features of specific glomerular circuits. Furthermore, this anatomical recovery of olfactory circuits allows recalling of odorant-dependent memories.

### Neuronal regeneration after methimazole injection

Different strategies have been used to ablate OSNs, such as surgical axon lesion, gas exposure, detergent irrigation, and systemic injection of toxins [4], [5], [36], [49], [50]. In every case, OSNs can regenerate in the OE and sensory axons maintain the ability to recreate circuits in the OB. At the OE level, the dorso-ventral organization of the different OSNs populations is completely restored [45]. However, these methods damage the LP and produce metaplasia of supportive tissue of the OE [5], [6]. At the OB, few axon fibers reach the dorsal part [5] and the specific glomerular organization is disrupted [36], [37].

In this study, we administrated methimazole systemically to ablate the OSNs. Previous studies have demonstrated that methimazole is metabolized by a cytochrome P450 in sustentacular cells and Bowman glands, causing massive apoptosis and promoting the degeneration of OE supportive cells [6], [51], [52]. This leads to detachment of OSNs while progenitor cells remain intact [53]. Contrary to other methods, methimazole lesions induce minimal damage to the LP with no evident metaplasia of the OE [6]. The structure of the LP has an important role in axon navigation in the olfactory system. Axon bundles pack together and run through the LP and they grow and innervate the OB [54].

Recent studies demonstrate that sensory axons are presorted before they reach the OB. This early organization is crucial for correct targeting of glomeruli [55]. Therefore, after an extensive damage, the structural integrity of the LP is essential for navigation of new axons. Here, we report that 45 days after methimazole administration the major features of M72 and I7 glomerular circuits are recovered. Most of the newly regrown axons innervate the dorsal and ventral aspects of the olfactory bulb and coalesce in few glomerular structures. Thus, it is possible that the main cause for the alterations in the pattern of projection reported in other studies is damage to the LP. Similar complete anatomical recovery of P2-olfactory glomeruli can be observed few weeks after inducing selective degeneration of P2-expressing sensory neurons, with no visible off-target projections [56]. However, our results show less robust recovery of OR-expressing OSN populations and more off-target axons. The context of massive regeneration occurring in our model could account for these differences.

Interestingly, we observe X-gal stained (M72) axons on the OB surface even were no OR-positive cellular bodies can be observed within the OE. Although we cannot rule out the possibility that some cells escaped ablation, our cell counting experiments suggest that these fibers are remnants of axons in which the degeneration process has not been completed. The fact that X-gal positive axons do no completely disappear from the olfactory system suggests that the process of neuron degeneration and recovery occurs so fast that new and remnant axons coexist in time. It is possible, therefore, that those residual axons could guide the rewiring process. This important issue must be addressed in future studies.

### Regeneration of glomerular structures

Previous studies have measured the variability of the glomerular position in several olfactory circuits [21], [57], [58], with variations ranging from 100 to 700 µm. Here we reported a variability of less than 150 µm of the M72 lateral glomeruli, after image normalization. This variability is consistent with other studies where the relative position is normalized with respect to a functionally identified glomerulus or adjacent tagged glomeruli [58], [21]. We focused on two aspects to determine the location of regenerated glomeruli: precision and accuracy. Precision reflects the intrinsic variation of projections of sensory neurons and the accuracy reflects the stereotypic position of glomeruli on the OB, reflected by the differences in location of regenerated glomeruli. We show that despite the increased number of glomeruli in the lateral hemibulb, there is no change in the precision and accuracy of glomeruli position after regeneration. This result strongly suggests that the mechanism that guides sensory fibers to their final location in the bulb is present in the adult mice and is similar to the one observed during development. Contrasting to postnatal development of glomerular circuits [46], refinement of regenerated circuits of adult mice is absent even 90 days after methimazole administration. Furthermore, the innervation of I7 glomeruli after regeneration appears irregular and possibly heterogeneous, i.e. it is likely that fibers from different OSN populations co-exist in some regenerated glomeruli, similarly to the initial steps in its formation during normal development [46]. Overall, our observations demonstrate that some mechanisms of axonal navigation and targeting persist from development to adult stage of the murine olfactory system; while others, chiefly those determining circuitry refinement, are absent.

### Odor memory and regenerated glomerular circuits

Previous studies have shown that olfactory function is recovered after regeneration of the olfactory system [38], [39], [59]. Discrimination capability recovers 15 days after lesion and the temporal course of learning of odorant-dependent task learning is identical between naive mice and those that have recovered from a lesion [38], [40]. However, regenerated animals are not able to recall a learned odorant-dependent task [38]. This phenomenon has been explained by alterations in the perceptual identity of odors produced by changes in the spatial organization of glomerular circuits.

Our results show that after methimazole administration, there is a remarkable restoration of the projection patterns of M72 and I7 circuits; which correlates with high success in recalling learned olfactory-dependent behavioral tasks, clued by the cognate ligands of M72 and I7 receptors (acetophenone and heptaldehyde, respectively). Although our anatomical analysis focuses only on M72 and I7 circuits, our behavioral data could suggest a similar degree of regeneration in other glomeruli. Moreover, the set of odorants used in this study has different molecular profiles and activates different regions along the OB: 2-heptanone activates the antero-dorsal [60], heptaldehyde the antero-ventral and dorso-medial regions of the bulb [61]. On the other odorant set, acetophenone [47] and citral [62] elicit responses in separate areas within the postero-dorsal bulb. Thus, it is likely that following the administration of methimazole the circuits responsible of recognition of all these odorants are reestablished as well.

Our results demonstrate that the disruption of glomerular organization on the OB observed in previous studies is the main cause of the loss of learned odorant-dependent behaviors after regeneration and underscore the importance of initial processing and segregation of olfactory information within the bulb for correct odorant identification and discrimination, which is necessary for the correct recall of memories associated with olfaction. Nevertheless, the behavioral tests used here differ from those used in previous studies and we cannot rule out that other subtle changes in olfactory performance occur, such as those observed with genetic models [47].

### Innate responses to odorants after regeneration

It has been shown that odorants can induce stereotypic behavioral responses in mice. During these responses, odorants are recognized simultaneously by the vomeronasal system, trigeminal and the main olfactory systems (i.e., the OE) [63]. It has been demonstrated that neural processing of aversive responses to TMT is performed by a set of glomeruli located specifically in the dorso-medial area of the OB. These circuits relay information to the bed nucleus of the stria terminalis (BNST), which is crucial for the repulsive behavior elicited by TMT [41]. Moreover, mice lacking the transient receptor potential channel (TRPC2), a crucial component in the sensory response of vomeronasal neurons, show no alterations in aversive response to TMT [64]. Therefore, aversion of regenerated mice to TMT suggests that the circuits responsible recover within the main olfactory system (OE and OB). However, the repulsive behavior elicited by TMT is altered in regenerated animals: mainly, the withdrawal response decreases significantly in comparison to control animals. These results could be explained to some degree by modifications in the centrifugal innervation of the OB or even to the BNST. Since it has been suggested that TMT could stimulate the trigeminal system [65], we cannot discard alterations to this system after methimazole exposure that could explain our results. Additional experiments are required to resolve this issue.

On the other hand, olfactory behavioral studies using female urine as an attractive clue for male mice demonstrate that the main olfactory system is determinant for its detection and for the elicited behavioral response [48]. Mice lacking the cyclic nucleotide gated channel (CNG2), crucial in the sensory response of OSN, disrupt the attractive and mating behavior elicited by urine, without altering the vomeronasal neurons response to it [66]. However, modifications in the organization of olfactory glomeruli in the main OB do lead to mating disruption [47]. Here we show that after regeneration there is no difference in the attraction response to urine (Fig. 7) or mating behavior (data not shown). Taken together, our results demonstrate that the specific glomerular organization in the OB is largely recovered after methimazole treatment.

In summary, we have demonstrated two important aspects of the olfactory system. First, the olfactory system has an enormous capacity to restore the general organization of the glomerular circuits after an extensive damage to the OSNs. Second, the organization of sensory inputs in the OB is important to determine the identity of odors and recovery of this organization is sufficient to recall memory traces associated with olfactory cues. Stability of these memory traces is not contingent upon persistent sensory input and can be reactivated when sensory input is re-established in the olfactory system.

## Materials and Methods

### Animals

Homozygous M72-IRES-tauLacZ and I7-IRES-tauGFP adult mice (6–9 weeks old) in a C57BL6×CBL5 genetic background were used for these experiments. All animals were maintained in a 12/12 light-dark cycle. Animal procedures followed the National Institutes of Health (NIH) guidelines for care and use of experimental animals (NIH approval number A5281-01). The protocols were revised and approved by the local animal rights committee of the Universidad Nacional Autónoma de México. Mouse strains were a generous gift from Dr. Peter Mombaerts.

### Ablation of olfactory sensory neurons and X-gal staining

A single intraperitoneal injection of methimazole (Sigma, 0.1 mg/g mouse weight) in vehicle solution (phosphate buffer –PBS- 0.1 M, 10% dimethyl sulfoxide –DMSO-) was administered to ablate the olfactory epithelium. Control animals received a single vehicle injection. For tissue preparation, mice were first anesthetized with sodium pentobarbital and perfused intracardially with cold PBS, followed by freshly prepared paraformaldehyde (PFA 4% in PBS). Whole-head mounts were post-fixed in cold PFA for 1 hr and dissected to expose either the medial aspect of the nasal turbinates or the dorsal aspect of the olfactory bulb. For coronal sectioning, whole-head mounts were post-fixed in PFA for 24 hr and then decalcified for 3–4 days in 30% sucrose PBS-DEPC 250 mM EDTA buffer. Serial slices of 20 µm were cut in a Leica cryostat CM1900. For X-gal (bromo-chloro-indolyl-galactopyranoside, Molecular Probes, Carlsbad, CA) staining, tissue was incubated for 5 hours in buffer containing 100 mM phosphate buffer (pH 7.4), 2 mM MgCl_2_, 0.01% sodium deoxycholate, 0.02% Nonidet P40, 5 mM potassium ferricyanide, 5 mM potassium ferrocyanide and 1 mg/ml of X-gal. Afterwards, the tissue was rinsed for 10 min in PBS (pH 7.4). Images were taken with a stereoscopic microscope Leica EZ4D and digitized with Leica FireCam software. Images were finally adjusted for brightness and contrast with ImageJ software (NIH) with no further manipulation.

### Cell counting

To analyze the temporal course of regeneration, M72-IRES-tauLacZ mice were sacrificed 1, 5, 10, 15, 20, 25, 30 and 45 days after methimazole administration (3–4 mice per group). Following X-gal staining in whole mounts, images from the medial aspect of the turbinates were obtained and X-gal stained cells were counted using the “Analyze Particles" plugin of ImageJ. Cells residing on the right and left part of the turbinates were counted independently and the average cell density (cells/mm^2^) was calculated. Images of the medial aspect of turbinates were filtered through contrast enhancement and background substraction before analysis. Cell counts in slices followed the same process as in whole mounts. One of every fourth section was collected up to a total of 29 slices. Only the olfactory epithelium was digitally selected in the images for counting procedures. For better comparison, control and experimental animals (45 days after methimazole) were sacrificed at the same age.

### Position analysis of olfactory lateral glomeruli

Images from the dorsal aspect of the olfactory bulb were obtained to determine the position of the lateral M72 glomerulus. Animals were grouped in control (n = 12) and methimazole-treated mice (n = 11). Images were taken at the same age in both groups, and were normalized to decrease variations in glomerular position related to the differences in bulb size. First, a rectangle delineating the medial, lateral, posterior and anterior edges was traced in each bulb. Then, all the rectangle sizes were averaged and normalized. Finally, the positions of centroids (x, y) of each glomerulus was determined and merged to the normalized plane size. To determine the dispersion of glomeruli, the standard distance was calculated as follows:
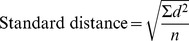
Where d is the Euclidean distance i.e. distance to a given point (x, y) from the average centroid position (x_m_, y_m_) and n is the total number of points. All image transformations were performed using ImageJ software.

### Immunochemistry

I7-IRES-tauGFP mice (n = 7, 3 control, and 4 experimental) were perfused as described above. After fixation, brains were cryoprotected in PBS+30% sucrose for 2 days at 4°C. Coronal OB slices (20 µm) were obtained. One of every third slice was collected. Sections were rinsed in 0.1 M PBS and incubated with a blocking solution containing 10% of normal horse serum and 0.3% Triton X-100 for 2 hours. Then, they were incubated with a polyclonal goat anti-GFP antibody (1∶1,000, Abcam) for 24 hours at 4°C. Finally, sections were rinsed and incubated for 2 hours with a donkey anti goat-Cy5 secondary antibody (Jackson InmunoResearch), washed and counterstained with fluorescent DNA stain DAPI (Invitrogen). Images shown in Figure 5 represent, in both cases, the ventro-lateral part of the OB of mice sacrificed at the same age. Sections were obtained approximately 500 µm after the beginning of their most anterior part. Images were obtained with a Leica DM6000 vertical microscope and digitized with Leica LAS AF software, and adjusted for brightness and contrast with ImageJ software.

### Behavioral tests

The olfactory discriminatory task performed by M72-IRES-tauLacZ male mice involved a three-armed maze and water-reward design. Mice were randomly divided in two groups. Group 1 had to discriminate acetophenone vs. citral and Group 2 heptaldehyde vs. 2-heptanone. During training, mice were restricted to a water intake of 1–2 ml per day. In the first 2 days of training, mice were habituated for 10 min to a three-arm maze in which water was delivered at the distal end of all three arms. Then for three weeks, one odorant of each pair was associated to the water reward. Odorant was delivered in a cotton ball (200 µl odorant/ball) placed in a hidden compartment on each arm. Two arms were selected to present the non-rewarded odorant (−) (citral or 2-heptanone) and one arm to present the rewarded odorant (+) (heptaldehyde or acetophenone). To minimize the effect of spatial learning, arms where randomly changed from day-to-day. In a training session of 10 minutes, mice explored the maze and water was delivered only in association with the conditioned stimulus in the +arm. During the first week of training, the water reward was delivered at a volume of 50 µl every time the animal made the right selection. The volume of reward was increased by 50 µl every week. To maintain a constant water intake of 1–2 ml per day, the percentage of rewarded trials was adjusted accordingly. After three weeks of training, we evaluated the performance of mice without delivering water (Test-Pre). For this, each mouse was placed inside the maze and behavior was recorded for 5 minutes. The videos were analyzed offline by a person without previous knowledge of the odorants used on each arm. The efficiency score for each arm was calculated by dividing the number of water rewarded entries during session by the total number of entries in that arm performed by each animal. For OE lesion experiments, 12 mice of each group showing efficiency scores ≥0.85 in the +arm and ≤0.35 in the −arm were selected. Group 1 and Group 2 were divided randomly into control (CTRL, n = 6) and experimental groups (EXP, n = 6). The EXP group was injected with methimazole two days after Test-Pre and the control group received a vehicle injection. The day of injection was designated as day 0. Two more evaluations were performed to follow the regeneration process at days 10 (Test 10-Post) and 45 (Test 45-Post). Fresh stock solutions of the odorants dissolved in DMSO were prepared every 10 days. Working solutions were dissolved daily in water to the following concentrations: 50 mM citral, 50 mM acetophenone, 10 mM heptaldehyde and 10 mM 2-heptanone. All odorants were purchased from Sigma.

Thirty-six mice were used for the innate preference test. To avoid learning, mice performed the task once and only for one odorant condition. Mice were habituated in a cage (27×17×13 cm) for 30 min, then the cage was replaced with a clean one and a piece of filter paper was introduced with fresh female urine (15 µl), trimethyl-thiazoline (TMT, 5 µl undiluted) or water (15 µl). Video recordings were taken and analyzed offline blindly to experimental condition. The investigation time was defined as approaches within 1 cm around the filter paper in a 3-minute period. The investigation time around purified water was established as the neutral response. Times greater and lower than the neutral response were interpreted as attraction or repulsion respectively [41]. The withdrawal index was calculated by dividing the number of abrupt changes in head orientation when approaching the filter paper by the total number of approaches.

### Statistical analysis

Graphs were generated with Excel and Origin lab 7. Statistical tests were performed in SYSTAT and GraphPad Prism 5 software. The Shapiro-Wilk test was done for each set of data to determine if they followed a normal distribution. Then we used parametrical or non-parametrical tests accordingly. For cell and mistargeted axon counts, we used parametrical tests (ANOVA followed by post hoc Tukey and T-student test were used, respectively). For glomeruli per hemibulb counts and innate behavior investigation times, we used non-parametrical tests (Kruskal-Wallis followed by post hoc Dunn and the Mann-Whitney, respectively). For non-parametric paired comparisons in olfactory discrimination data, we used the Wilcoxon test. Finally, glomerular positions were analyzed with two parametrical tests: the Levene test was used to evaluate differences on variance of the Euclidean distance of glomeruli position; then, as suggested by Levine [42], sets of coordinates were compared by analyzing both axis and correcting for multiple comparisons with Bonferroni correction test.
